# Ex Vivo and In Vivo Characterization of Interpolymeric Blend/Nanoenabled Gastroretentive Levodopa Delivery Systems

**DOI:** 10.1155/2017/7818123

**Published:** 2017-04-26

**Authors:** Ndidi C. Ngwuluka, Yahya E. Choonara, Girish Modi, Lisa C. du Toit, Pradeep Kumar, Leith Meyer, Tracy Snyman, Viness Pillay

**Affiliations:** ^1^Wits Advanced Drug Delivery Platform Research Unit, Department of Pharmacy and Pharmacology, School of Therapeutic Sciences, Faculty of Health Sciences, University of the Witwatersrand, Johannesburg, 7 York Road, Parktown 2193, South Africa; ^2^Department of Neurology, Faculty of Health Sciences, University of the Witwatersrand, Johannesburg, 7 York Road, Parktown 2193, South Africa; ^3^Department of Paraclinical Sciences, Faculty of Veterinary Science, University of Pretoria, Pretoria, South Africa; ^4^National Laboratory Services, Faculty of Health Sciences, University of the Witwatersrand, Johannesburg, 7 York Road, Parktown 2193, South Africa

## Abstract

One approach for delivery of narrow absorption window drugs is to formulate gastroretentive drug delivery systems. This study was undertaken to provide insight into in vivo performances of two gastroretentive systems (*PXLNET *and IPB matrices) in comparison to Madopar® HBS capsules. The pig model was used to assess gastric residence time and pharmacokinetic parameters using blood, cerebrospinal fluid (CSF), and urine samples. Histopathology and cytotoxicity testing were also undertaken. The pharmacokinetic parameters indicated that levodopa was liberated from the drug delivery systems, absorbed, widely distributed, metabolized, and excreted. *C*_max_ were 372.37, 257.02, and 461.28 ng/mL and MRT were 15.36, 14.98, and 13.30 for Madopar HBS capsules,* PXLNET*, and IPB, respectively. In addition, X-ray imaging indicated that the gastroretentive systems have the potential to reside in the stomach for 7 hours. There was strong in vitro-in vivo correlation for all formulations with *r*^2^ values of 0.906, 0.935, and 0.945 for Madopar HBS capsules,* PXLNET*, and IPB, respectively. Consequently,* PXLNET *and IPB matrices have pertinent potential as gastroretentive systems for narrow absorption window drugs (e.g., L-dopa) and, in this application specifically, enhanced the central nervous system and/or systemic bioavailability of such drugs.

## 1. Introduction

Although a number of in vitro drug delivery studies are undertaken, the ultimate goal in developing and evaluating a drug delivery device is to achieve the desired drug delivery outcomes in vivo. Despite attempts at simulating in vivo environment, in vitro studies still do not exactly replicate the operation and impact of an in vivo environment on drug delivery devices. Hence, after development and in vitro analyses, there is still the need to assess the device in vivo (within a living organism) before it is commercialized for administration to the end-user consumer for complete description of the pharmacodynamics and pharmacokinetics data of the drug delivery device. The degree of absorption of a drug in the gastrointestinal tract is based on certain events which include drug release, drug in solution at absorptive sites, drug absorption into systemic circulation, liver and gut metabolism, decomposition, and transit [1]. Absorption and subsequent bioavailability of a drug are not only determined by the properties of the drug, such as solubility, which in turn is based on its crystallinity and lipophilicity, but are also affected by the gastrointestinal environment which is determined by its pH and presence of food and certain substances such as surfactants in gastric juice or bile as well as enzymes. Other factors include viscosity of luminal contents, motility patterns and flow rate, secretions and coadministered fluids [2]. Hence, oral drug delivery devices are developed to accommodate a number of these factors and at the same time ensure the absorption and subsequent bioavailability of the incorporated drug.

Optimized interpolymeric blend/nanoenabled levodopa- (L-dopa-) loaded delivery systems have been developed to be gastroretentive and release levodopa at a constant rate in order to maintain a constant concentration over a prolonged period for potential in vivo attainment of enhanced bioavailability of the narrow absorption window L-dopa. Hence, it was necessary to assess in vivo the gastric residence time and drug release properties, as well as the degree of toxicity of the device. The pig model was chosen because of the close resemblance of its gastrointestinal tract to that of humans, consequently, being best suited for in vivo studies of oral drug delivery. The anatomy and physiology of each section of the pig's gastrointestinal tract are comparable to that of humans [3, 4]. The pig model has also been employed to model brain disorders. This is due to the similarities of the pig's brain to that of a human in extent of peak brain growth at the time of birth, the gross anatomy, and the growth patterns [5, 6]. The catecholaminergic neurons in the pig brain are similar to those in other vertebrates [6]. Furthermore, a study by Minuzzi and coworkers indicated that the saturation binding parameters (*B*_max_ and *K*_*d*_) of ligands specific for dopamine D_1_ and D_2_ receptors in pig brain cryostat sections are similar to the human receptors [7] implicated in the pathophysiology of Parkinson's disease [8]. Consequently, the choice of a pig model was deemed appropriate to assess the in vivo performance of L-dopa-loaded gastroretentive delivery systems.

## 2. Materials and Methods

### 2.1. Materials

The following materials were sourced for cell and animal studies: heparin sodium 1000 i.u./mL (Bodene (PTY) Limited as Intramed, Port Elizabeth, South Africa), normal saline (Adcock Ingram, Midrand, South Africa), two-lumen central venous catheterization set with ARROWgard Blue (Arrow International, Inc., Reading, PA, USA), CaCo 2 adhesion cells, CytoTox-Glo™ Kit (Promega Corporation, Madison, WI, USA) fetal bovine serum, penicillin and streptomycin, Dulbecco's modified Eagle's medium (DMEM) (Sigma-Aldrich Chemie, GmbH, Steinheim, Germany), acid washed alumina, TRIS buffer, phosphoric acid (Bio-Rad Laboratories, Hercules, CA, USA), Oasis® HLB cartridges (3cc, Waters Corporation, Milford, MA, USA), silicone Foley catheters (two-way French size 10, Supra Latex, Kempton Park, Gauteng, South Africa), clinical speculum and veterinary laryngoscope, levodopa, dopamine, methyldopa, benserazide, and carbidopa (Sigma-Aldrich Chemie, GmbH, Steinheim, Germany). Materials used for formulation of the tablet matrices were methacrylate copolymer (Eudragit E100, Evonik Röhm GmbH & Co. KG, Darmstadt, Germany), sodium carboxymethylcellulose (NaCMC, Fluka Biochemika, Sigma-Aldrich Chemie, GmbH, Buchs, Switzerland), locust bean from* Ceratonia siliqua* seeds (Sigma-Aldrich, Inc., Steinheim, Germany), barium sulphate, pullulan from* Aureobasidium pullulans* (Sigma-Aldrich, Inc., Steinheim, Germany), silica, magnesium stearate (Merck Chemicals (Pty), Ltd., Gauteng, South Africa), chitosan (Wellable Group, Fujian, China), sodium tripolyphosphate (TPP) (Sigma-Aldrich, Germany), and lecithin from egg yolk (Lipoid E PC S, Lipoid AG, Ludwigshafen, Germany).

### 2.2. Preparation of Gastroretentive Formulations

The formulations* Poly-x-Lipo Nanoenabled Tablets (PXLNET) *and interpolymeric blend (IPB) matrices were prepared as previously described, where the IPB had L-dopa and the decarboxylase inhibitor, benserazide, directly compressed into the matrix, whereas the* PXLNET* L-dopa and benserazide were incorporated into the IPB matrix within poly-lipo-nanoparticles [9, 10]. However, the* PXLNET* was modified in this study for easy administration to the pigs. A tablet of not more than 1000 mg in total was permitted due to the method of administration. Consequently, the quantity of IPB was reduced to 224.22 mg, while the quantity of levodopa-loaded nanoparticles was 375.78 mg. Madopar HBS, a controlled release as well as a gastroretentive dosage form, was employed to analyze the performance of IPB and* PXLNET *gastroretentive drug delivery systems.

### 2.3. Arrival of Pigs and Habituation

The animal ethics clearance (2009/01/05) was obtained from the animal ethics screening committee of University of the Witwatersrand, Johannesburg, South Africa. Five White Large pigs (four females and a male) weighing 32.55 ± 4.38 kg were used for the study. The pigs were housed in cages with access to food and water under a controlled temperature (20–24°C) and a 12-hour light/dark cycle. Habituation was ensured before the pigs were subjected to surgery and dosing.

### 2.4. Venous Catheterization of the Pigs for Blood Sampling

Approximately ten days after arrival, surgery was undertaken under aseptic conditions to insert a catheter in the internal jugular veins of the pigs for easy withdrawal of blood samples during dosing. Briefly, each pig was anesthetized with ketamine (11 mg/kg) and midazolam (0.3 mg/kg) intramuscularly and maintained by intubation with 2% isoflurane in 100% oxygen. Analgesia was provided by intramuscular administration of buprenorphine (0.05 mg/kg) and carprofen (4 mg/kg). An incision was made on the lateral side of the neck to expose the jugular vein, which was isolated and a two-lumen central venous catheter was inserted into the lumen of the vein. The remainder of the catheter was tunneled subcutaneously with the aid of a trocar to an exit point cranial to the dorsal aspect of the scapular. To avoid untimely removal of the catheter by the movements of the pig, the external sampling ports were sutured to the skin of the pig. The catheter was tested and cleaned by withdrawal of blood and flushing with heparinized saline (5000 i.u./L of 0.9% saline). The pigs were monitored after surgery to ensure full recovery from anesthesia and allowed more than seven days to recover before the commencement of gastric dosing and sampling.

### 2.5. Flushing and Bleeding of Pigs

In order to keep the catheters open throughout the period of the study, the catheters had to be flushed twice a day with heparinized saline. Bleeding was also undertaken at intervals to ensure flow of blood through the catheters as well as to obtain blank plasma. In addition, the ports of the catheters had to be sprayed with antiseptic before and after flushing to avoid infection.

### 2.6. Gastric Dosing and Blood Sampling of the Pigs

**Figure 1:**
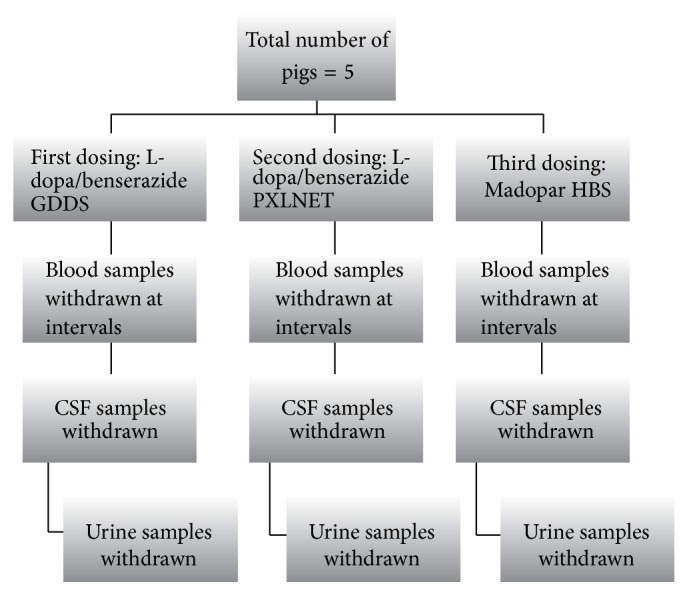
Flow diagram detailing in vivo animal studies for three drug delivery systems.

The pigs were fasted overnight before dosing. The formulations* PXLNET *and IPB matrices as well as Madopar HBS were administered via intragastric tubes. However, before dosing, baseline blood samples were withdrawn for control analysis. The procedures for flushing and bleeding were utilized to withdraw the baseline blood samples and subsequent blood samples from the pigs after dosing. The pigs were anesthetized as described earlier and subsequently each pig was raised in an upright position and, with the aid of an intragastric tube, the drug was administered via the tube and flushed down into the stomach with about 20–50 mL of water. The pigs are taken back to their cages and monitored until they recovered from anesthesia. The study was a crossover study with two-day wash-out period, whereby the same pigs were employed for the different dosage forms. Blood samples were withdrawn from the chronically implanted venous catheters at specific time intervals (2, 4, 6, 8, 10, 12, 16, 20, and 24 hours) and collected in EDTA vacutainers (BD Vacutainers®, Franklin Lakes, NJ, USA) to avoid coagulation. The blood samples were centrifuged at 5000 rpm for 15 mins to obtain plasma samples. Into 2 mL of each plasma sample, 30 uL of 10% sodium metabisulphite was added and the plasma samples were stored in a −80°C freezer until analysis.  Figure 1 explicates the crossover design and the dosage forms administered.

### 2.7. Cerebrospinal Fluid Collection from Pigs

Cerebrospinal fluid (CSF) was obtained from an anaesthetized pig by puncturing the cisterna magna. The cistern magna can be accessed through the foramen magnum. The pig's neck was leaned on the table to flex the neck by an assistant. The caudal end of the occipital bone and the nuchal tubercles were palpated. A 20-gauge spinal needle was passed slightly caudal to this area at an angle approximately 60° towards the oral cavity to enter the foramen magnum cranial to the body of the axis. CSF was withdrawn with a 2 mL syringe and transferred into a collection tube containing 10% sodium metabisulphite. The CSF sample was then stored at −80°C until analysis. CSF was collected at the 2nd and 4th hour after dosing. CSF sampling was not carried out over the day because the pigs could only be anaesthetized a limited number of times in a day and puncturing of the cisterna magna was also limited.

### 2.8. Urine Collection from Pigs

The pigs were anaesthetized and placed on their abdomens. A lubricated speculum with a long blade was inserted into the urogenital opening to open the vaginal wall. To visualize the external urethral orifice, a veterinary laryngoscope with straight blade was inserted. The female urethral opening is located on the floor of the vagina, about a third or half the distance to the cervix. A Foley catheter French size 10 with a stylet was controlled with a blunt tip forceps and inserted into the bladder. As the catheter got into the bladder, the stylet was removed and urine was allowed to flow into the collection tube. Urine was collected at the 2nd and 4th hour after dosing. More time point urine sampling was limited due to the same reasons for CSF collection. This study adhered to the scope of approval by the animal ethics committee.

### 2.9. In Vivo Measurement of the IPB GDDS and* PXLNET* Residence Times in a Large White Pig Model

Measurement of the gastric residence time of a drug delivery system at the application site is to provide information on the gastroretentive ability of the drug delivery system. X-ray imaging was employed as a noninvasive method of determining the residence time without affecting gastrointestinal (GIT) motility. A radio-opaque marker, barium sulphate, was incorporated into the GDDS and* PXLNET* formulations to determine the extent of gastroretention. Two of the Large White pigs were fasted overnight and a radiolabeled GDDS and* PXLNET* was administered to them on different occasions. The animals were anaesthetized twice: first, it was during drug delivery system administration and, second, at the 7th hour after administration, to undergo X-ray imaging each time point.

### 2.10. Histopathological Evaluation in Control and Dosed Pigs

The stomach of a euthanized pig was cut open and the area the PXLNET was located, was excised, as well as the posterior and anterior section, and was fixed in neutral buffered formalin. The same sections were excised from the control pig and fixed in neutral buffered formalin in order to preserve the tissues. The tissue samples were embedded on labeled cassettes and sectioned into blocks. An automated processor was used for fixation, dehydration, and paraffin embedding. Routine histological methodology was undertaken which involved Mayer's hematoxylin and eosin staining procedure. Coverslipping was undertaken to prevent the tissue from being scratched and to provide better optical quality during microscopic viewing. Descriptions of the microscopic features were made and a final microscopic diagnosis was reported.

### 2.11. Cytotoxicity Testing of the IPB and Nanoparticles

**Figure 2:**
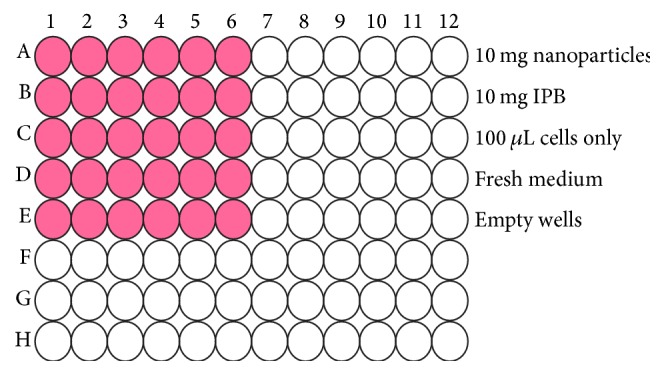
Schematic diagram of a 96-well plate depicting the arrangement of the samples, no-cell background and cells only.

CaCo-2 adhesion cells were cultured in 10 mL cocktail media comprising 10% fetal bovine serum (5 mL), 0.1% v/v of penicillin (100 IU/mL) and streptomycin (100 *μ*g/mL), and Dulbecco's modified Eagle's medium (DMEM). The cells were maintained in a humidified atmospheric incubator (RS Biotech Galaxy, Irvine, UK) with 5% CO_2_ at 37°C. The cells were cultured under aseptic conditions to avoid contamination and death. After growing the cells for two weeks, the medium was decanted and the adherent cells were rinsed with DMEM. Thereafter, the adherent cells were harvested by trypsinization (100 *μ*L trypsin was added and incubated for 5 minutes). The cells were washed with fresh medium (DMEM) to remove residual trypsin and resuspended in fresh medium. The suspended cells (100 *μ*L each) were placed in a 96-well plate as shown in Figure 2 and 10 *μ*L of samples (0.1 mg/*μ*L) was added to each of the wells containing cells. The colored wells as shown in Figure 2 contain cells and samples tested. The 96-well plate was incubated for 24 hours.

After 24 hours of incubation, the cytotoxicity assay was performed employing CytoTox-Glo Kit (Promega Corporation, Madison, WI, USA). CytoTox-Glo cytotoxicity assay is a homogenous luminescent assay which enables the number of dead cells in a well to be counted. The assay has two steps: first is the addition of the luminogenic peptide substrate which enables the measurement of dead-cell protease activity released from cells that have lost membrane integrity, and the second step requires the addition of the lysis reagent to deliver a luminescent signal associated with the total numbers of cells in each well. The number of dead cells was measured at each step after 15 mins incubation at ambient temperature by a multilabel reader (PerkinElmer 2030 Victor™, Turku, Finland).

### 2.12. Ultraperformance Liquid Chromatographic Analysis of Samples

#### 2.12.1. Quantitative Analyses of Samples

Quantitative assays of samples were performed on Waters Acquity™ UPLC/MS/MS system (Waters Corporation, Milford, MA, USA). The column used was an Acquity UPLC® BEH shield RP18 1.7 *μ*m, 2.1 × 100 mm. Carbidopa was used as internal standard and a gradient method was employed using mobile phase, 2 mmol/L ammonium acetate and 0.1% formic acid in deionized water as solvent A and acetonitrile as solvent B. The ratio of the mobile phase gradient started at 30% A for 0.5 min and increased linearly to 100% B for 1 minute, returning to the original settings over the following 0.5 min at a flow rate of 0.3 mL/min. The injection volume was 10 *μ*L, run time was 2 min, and sample temperature was maintained at 4°C. The data was captured with Waters MassLynx™ software. Standards and analytes were detected using a triple quadrupole mass spectrometer fitted with electrospray ionization probe (ES+) and multiple reaction monitoring scan; parameters are as shown in Table 1.

#### 2.12.2. Standard Preparation of Actives

Stock solutions of L-dopa, dopamine, methyldopa, benserazide, and carbidopa were prepared by dissolving 100 mg of each drug in 100 mL of 0.1 N hydrochloric acid individually. From the stock solutions, a series of working standards was prepared in blank plasma to give 4000, 2000, 1000, 500, 250, and 125 ng/mL each of L-dopa, dopamine, methyldopa, and benserazide combined in each working standard, while carbidopa added to the standards was 2000 ng/mL to provide a standard curve required for quantitation. Extraction from plasma was undertaken before injection and a standard curve was obtained from the peak ratio of drug/internal standard versus the concentrations of standards. The curve type is linear with a weighting factor of 1/concentration.

#### 2.12.3. Extraction of Drugs and Metabolites from Plasma and CSF Samples

Frozen plasma and CSF samples were thawed and 2 mL of each sample was transferred into separate extraction tubes. A designated measuring spoon was used to add one level spoonful of alumina into each tube. Thereafter, 2000 *μ*L of the internal standard, carbidopa, was added, followed by 1 mL of TRIS Buffer. The tubes were capped and agitated using a mechanical shaker for 5 min. The tubes were centrifuged at 2500 rpm for 2 min. A disposable pipette was used to remove as much liquid as possible from each tube without disturbing the alumina. To wash the alumina, 1 mL Milli-Q water was added to each tube; the tubes vortexed for 15 secs and centrifuged for 2 min at 2500 rpm. Water was removed using disposable pipettes. The washing procedure was repeated and 200 *μ*L of 0.1% phosphoric acid was added to the tubes and vortexed for 30 secs. The tubes were centrifuged for 2 min at 2500 rpm and the supernatant was transferred into sample vials for subsequent injection into the column.

#### 2.12.4. Extraction of Drug and Metabolites from Urine

Solvent-phase extraction was employed to isolate metabolites from urine. Briefly, 2 mL of methanol was used to condition each of the Oasis HLB cartridges and 2 mL of deionized water was used for washing. Thereafter, 2 mL of urine sample was loaded onto each cartridge, followed by 2 mL of 5% methanol in water. The metabolites were then eluted with 500 *μ*L methanol and acetonitrile in the ratio of 1 : 1. The eluates were then transferred into sample vials for subsequent injection into the column.

### 2.13. Pharmacokinetic Modelling and Analysis

PKSolver, an add-in program for Microsoft Excel written in visual basic for application (VBA) for decoding problems in pharmacokinetic and pharmacodynamic data analysis, was used to model and estimate the pharmacokinetic parameters. Analysis of variance (ANOVA) was used to determine the statistical significance of the differences between the data.

## 3. Results and Discussion

Venous catheterization was successful. The pigs healed as anticipated without infection and dosing commenced. There was successful blood sampling at time intervals as well as CSF withdrawals. Urine collection was not as successful in all pigs on all days of dosing and sampling. One of the pigs had a skewed urethra and three attempts on different days proved abortive. In another, the urogenital canal began to bleed during the process and urine was not sampled from the pig.

### 3.1. In Vivo Measurement of the GDDS and* PXLNET* Residence Times in a Large White Pig Model

**Figure 3:**
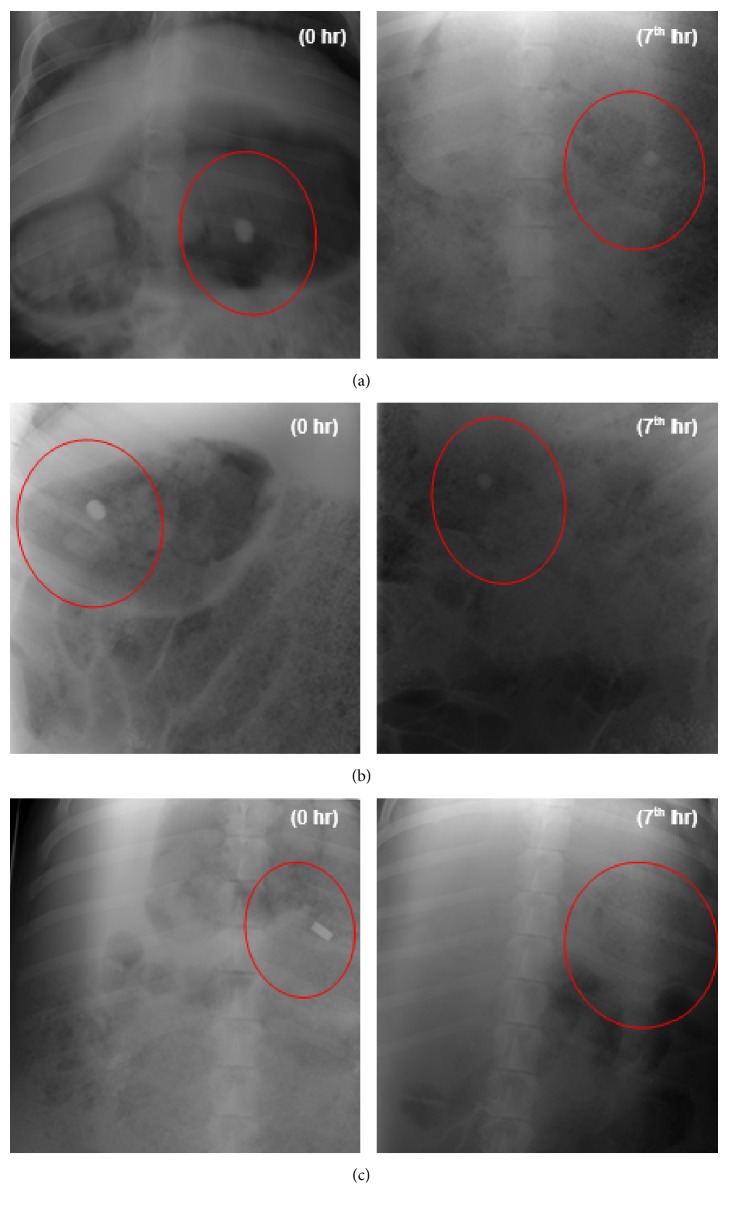
Radiographic images of (a) GDDS with the pig in the anterior-posterior position; (b) GDDS with the pig in the lateral position; and (c) PXLNET with the pig in the anterior-posterior position.

Two pigs were utilized for the in vivo gastroretentive study and the radiographic images were captured at the lateral and anterior-posterior positions as shown in Figure 3. The images in Figure 3(a) are the anterior-posterior position of the pig showing the presence of the device in the stomach immediately after dosing and at the 7th hour indicating that the IPB GDDS is able to be retained in the stomach for at least 7 hours. The position of the GDDS can be found within the red circles on the images. The radiographic images at the 7th hour showed that GDDS retained its three-dimensional network. However, the presence of the GDDS could not be seen in the second pig. It is envisaged that GDDS could have been obscured by food as the pigs were allowed to eat after administration and recovery from anesthesia or it could have been emptied from the stomach which may be an indication of intersubject variability.

However, as observed during in vitro drug release studies, PXLNET lost its three-dimensional network due to more rapid erosion in the presence of fluid [9] and may be showing as dispersed particles faintly seen in Figure 3(c) within the red circle. Furthermore, when a dosed pig was euthanized to harvest the stomach for histopathological testing 4-5 hours after administration, PXLNET was found adhering to the wall of the stomach perhaps kept in place by the presence of food but it had lost its shape. This is indicative that PXLNET may be able to withstand peristalsis up to 5 hours.

### 3.2. Histopathological Findings in Dosed and Control Pigs

**Figure 4:**
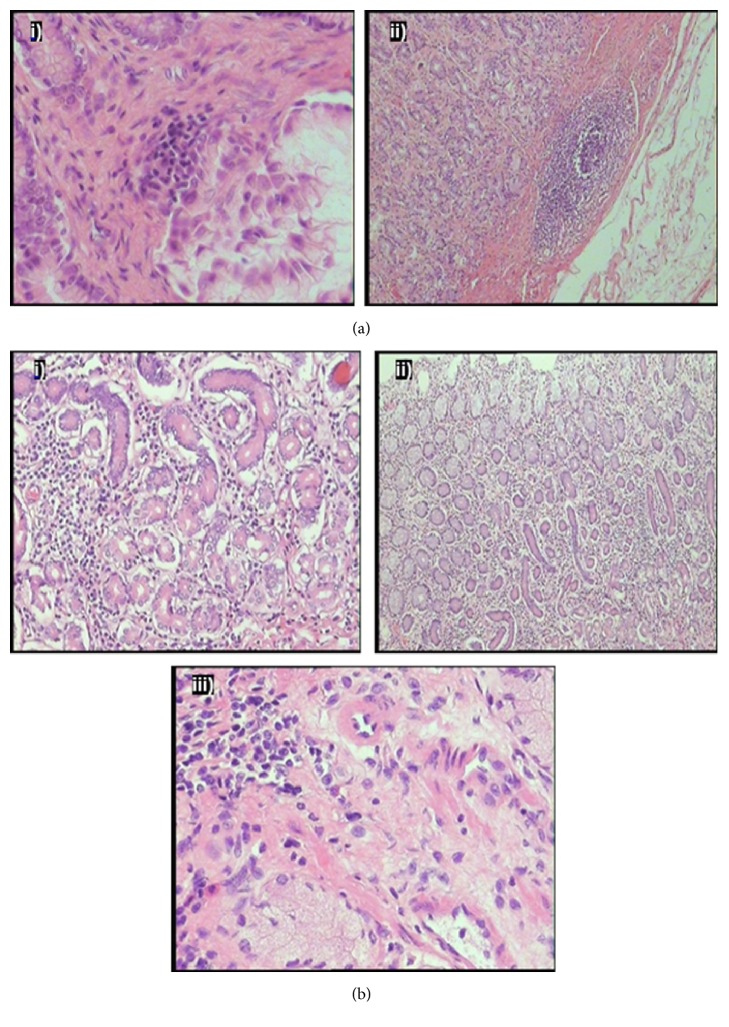
(a) Images from dosed pigs' stomach showing (i) mild lymphocytic aggregate in lamina propria interstitium and (ii) lymphoid follicle in deep lamina propria and submucosal edema. (b) Images from control tissue: (i) moderate lymphoplasmacytic interstitial lamina propria infiltration, higher magnification (×20); (ii) moderate lymphoplasmacytic interstitial lamina propria infiltration, lower magnification (×10); (iii) mild lymphoplasmacytic interstitial aggregated in the lamina propria.

Histopathological findings for the dosed (either with IPB or PXLNET) and control pigs are shown in Figure 4.

#### 3.2.1. Dosed Animal

The mucosal epithelium was multifocally lost, likely due to autolytic changes of an early degree. The gastric glands appeared normal. Few normal appearing lymphoid follicles were visible in some sections, within the muscularis mucosa. The submucosa in few areas appeared mildly edematous. Very few lymphoplasmacytic aggregates were present in the lamina propria interstitium, mostly in one of the biopsy specimens from the pyloric area of the stomach wall.

#### 3.2.2. Control

The stomach mucosal epithelium was multifocally lost, likely due to early autolytic changes. Where intact, the mucosal epithelium appeared normal with mucus accumulation together with intact desquamated epithelium cells on the surface. The underlying lamina propria multifocally showed mild lymphocytic infiltrates. These infiltrates extended to the muscularis mucosa but not beyond that. The gastric glands appeared within normal limits. Samples from both the fundus and pyloric portions of the stomach wall were available for examination. One section from the pylorus revealed moderate interstitial inflammation in which lymphocytes, plasma cells, and eosinophils were all present in a mixed reaction. The submucosa appeared mildly edematous.

The control sample yielded more inflammatory changes in the stomach lamina propria than the dosed sample. Mild inflammation was, however, present in dosed and control pigs and changes can therefore not be related directly to the polymeric drug delivery system used in the dosed pig. Mild gastric inflammation is a nonspecific lesion in many production animals and may be related to intestinal flora, intestinal pathogens, and presence of worms.

### 3.3. Cytotoxicity Testing of the IPB and Nanoparticles

**Figure 5:**
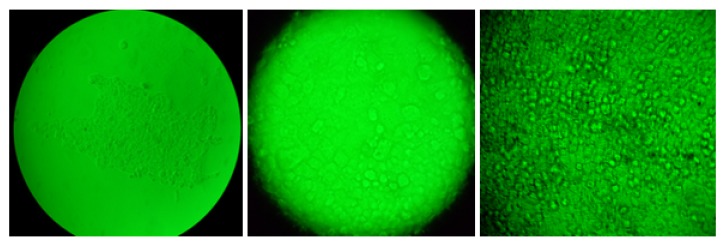
Microscopic images of CaCo-2 adhesion cells.

The results obtained from the cytotoxicity testing are shown in Tables 2–5.  Table 5 shows the percentage cytotoxicity for all samples. The luminescent signals observed for fresh medium and empty wells were used to correct those obtained for the samples and the percentage cytotoxicity was calculated thereafter. The cytotoxicity data obtained indicated that the drug delivery devices were not cytotoxic. This is not unexpected as the polymer utilized such as sodium carboxymethylcellulose [11–13] and chitosan [14–16] have been found to be cytoprotective. While studies on the cytoprotective nature of locust bean could not be obtained, it is generally regarded as safe (GRAS).  Figure 5 shows confocal microscopy images of the cells viewed during culturing.

### 3.4. UPLC/MS/MS Method Validation: Recovery, Linearity, and Limit of Detection

Efforts have been made to optimize the quantitation of L-dopa, benserazide, and the metabolites. However, catecholamines are in the submicroanalysis range, a few parts per billion in the plasma; further, they are to be extracted from complex biological systems such as plasma, which usually poses a challenge of obtaining sufficient yields [17]. The recovery of the drugs was assessed by comparing the area under the curves and peak heights of the standards extracted from the plasma to those in aqueous solutions and of the same concentrations. Percentage recovery ranged from 82 to 122% for methyldopa, 89 to 125% for dopamine, and 81 to 114% for L-dopa at concentration ranges from 125 to 8000 ng/mL. The limit of detection is described as the concentration of the analyte that produces a signal equal to three times the standard deviation of the signal from the blank. The error limit of detection is calculated as 3 times the standard deviation obtained from the blank or as 3 times the height of the baseline of the blank. The limit of detection was 40.60 ng/mL, 85.69 ng/mL, and 54.94 ng/mL for methyldopa, dopamine, and L-dopa, respectively. Specificity is derived from the mass selectivity and multiple reaction monitoring transitions, while linearity is related to correlation coefficients ranging from 94 to 99% for methyldopa, 86 to 97% for dopamine, and 96–99% for L-dopa.

### 3.5. Pharmacokinetic Data Analysis

Pharmacokinetic analysis is crucial in order to assess the in vivo performance of a drug delivery system. Before a drug is orally absorbed, it has to be liberated from its carrier. The factors that influence the oral absorption are broadly categorized as biological factors, physiochemical properties of the drug, and formulation factors. These factors influence the pharmacokinetic phase of drug administration and so determine the drug level in the systemic circulation, site of action, and subsequently the therapeutic effect of the drug administered.

**Figure 6:**
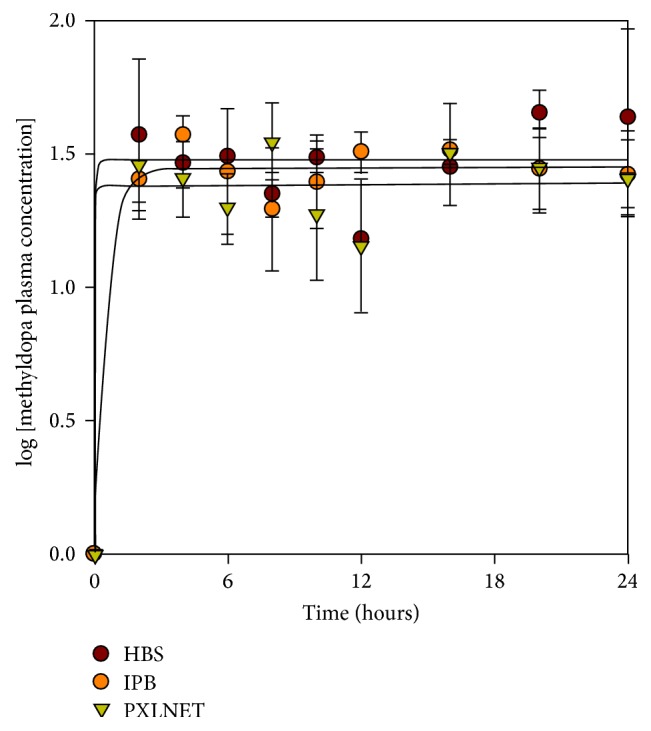
Mean methyldopa plasma concentration after administration of Madopar HBS capsules and* PXLNET* and IPB matrices.

**Figure 7:**
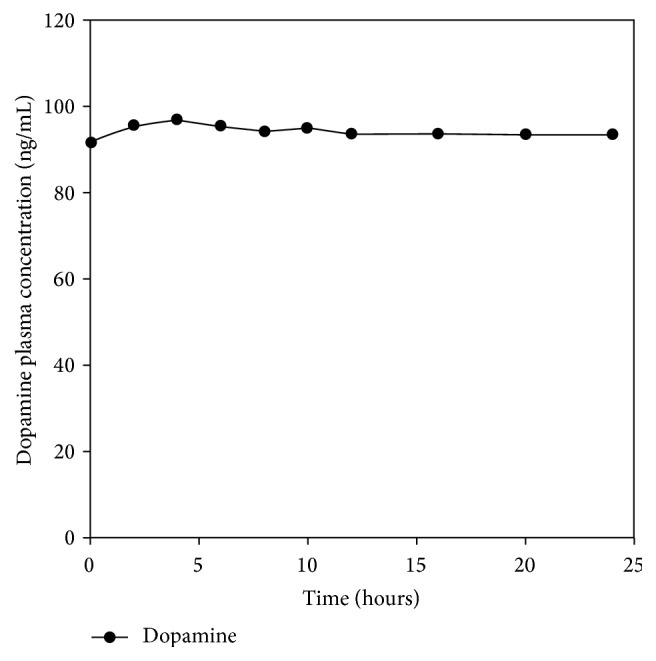
A typical mean dopamine plasma concentration observed for all formulations.

**Figure 8:**
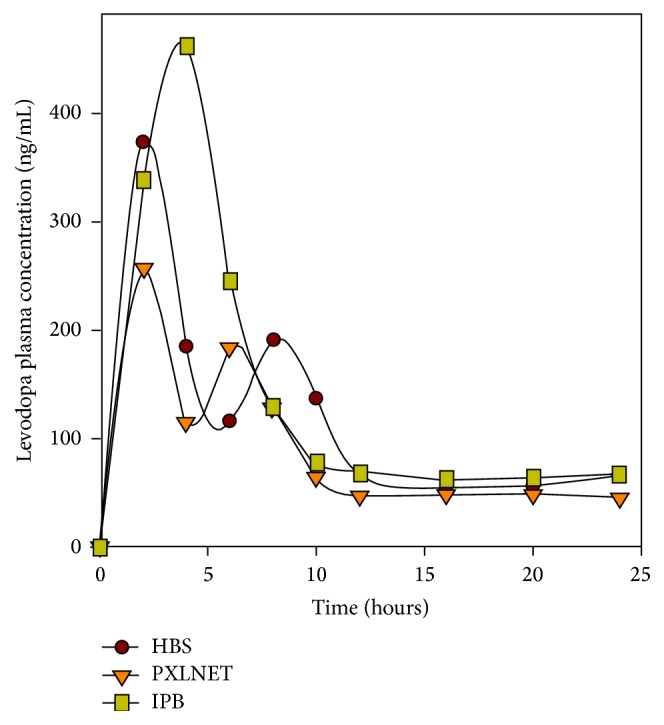
Comparative L-dopa pharmacokinetic curve of Madopar HBS capsules and* PXLNET* and IPB matrices after single dose administration.

The pigs fared well after administration of drug and recovery from anesthesia, though two pigs took a longer time to recover from the effect of anesthesia and puncturing of the cisterna magna. On visual observation, they did not seem to exhibit any side effects associated with administration of L-dopa. The metabolites and L-dopa plasma concentrations are presented in Figures 6, 7, and 8, while CSF and urine concentrations are shown in Tables 6 and 7, respectively; and the pharmacokinetic parameters are presented in Table 8. Benserazide was not detected in the samples. Benserazide, as observed during the study and confirmed in the literature, is highly chemically unstable, making its analytical quantitation challenging. It is rapidly metabolized to its main metabolite trihydroxybenzylhydrazine, a highly potent decarboxylase inhibitor [18]. Jorga and coworkers [18] also could not measure benserazide in some of the patients used in their study. They observed an increase in benserazide levels in patients given a 50 mg dose. In this study, 25 mg of benserazide was administered to the pigs. Furthermore, Jorga and coworkers [18] observed that the metabolite trihydroxybenzylhydrazine was rapidly formed after the administration of benserazide and its concentration exceeded that of benserazide. Although benserazide was metabolized, the presence of its metabolite, trihydroxybenzylhydrazine, ensured continued carboxylase inhibition leading to the presence of unchanged L-dopa in the urine and no significant difference in the level of dopamine plasma concentration.

The plasma concentration of methyldopa was observed to vary between 8 and 50 ng/mL in the three formulations (Figure 6) and there was no marked increase over the period of sampling. Nutt and coworkers also observed that each dose of L-dopa probably made a small contribution to the plasma concentration of methyldopa [19]. They deduced that methyldopa fluctuations observed in the plasma concentrations may be due to redistribution within the tissues. Furthermore, the concentrations of methyldopa in the plasma in comparison to other large neutral amino acids suggest that it is not a major competitor with L-dopa for transport to the brain; and, hence, at the concentrations detected during L-dopa dosing, it is not an important determinant of clinical response [20].

In the absence of a carboxylase inhibitor, more than 90% of L-dopa is converted to dopamine [21]. In this study, dopamine was essentially constant in all the formulations and confirms the effective carboxylase inhibition by benserazide/its active metabolite. A typical dopamine plasma concentration-time curve is shown in Figure 7. Dopamine was not detected in most of the CSF samples (Table 6). However, there is a noted dopamine concentration in the CSF for the PXLNET system. This is possibly due to enhanced targeted delivery of both L-dopa and benserazide attained by the nanoenabled system due to intact nanoparticles potentially achieving more site-specific conversion of L-dopa to dopamine. Olanow and coworkers also could not detect free dopamine in CSF [22]. Furthermore, the large presence of dopamine in the urine (Table 7) did not stem mainly from L-dopa dosing. The bulk of the urinary dopamine may be from renal production and uptake of dopamine and decarboxylation of circulating dihydroxyphenylalanine (dopa), [23, 24] which is in turn from hydrolysis of tyrosine. However, the rationale for the large concentration of dopamine at the 4th hour for Madopar HBS in comparison to* PXLNET *and IPB matrices is uncertain. It is known that urinary dopamine is increased by feeding [25] and stress [26] amongst other factors.

A comparative display of L-dopa concentration-time curves for the three formulations is provided in Figure 8. The pharmacokinetic curves and parameters obtained for each formulation are dependent on the rate of release of L-dopa from the formulation and biological factors such as health/disposition of GIT, gastric emptying rate, rate of absorption, rate of metabolism, transporters, and extent of distribution, amongst other factors. These factors are expected to vary from pig to pig (intersubject variation) and it is also possible to vary within a pig over time (intrasubject variation). Furthermore, a protein-loaded diet is known to decrease the oral absorption of L-dopa. This is due to competitive absorption in the presence of proteins as L-dopa uses the same transport system as large amino acids. However, it is also been found that food effects vary with formulations [21].

In addition, double peaks observed in the pharmacokinetic curves of Madopar HBS capsules and* PXLNET *matrices may be attributed to the effect of L-dopa on gastric emptying time. Studies have shown that L-dopa produces intermittent delays in gastric emptying time [27–29]. In the studies undertaken by Robertson and coworkers, the double peaks were shown to correspond to the two distinct phases of gastric emptying separated by a period of negligible or no significant emptying. They employed paracetamol which is a biomarker for gastric emptying with radiolabeled diethylenetriaminepentaacetic acid (^99^Tc-DTPA) and gamma-camera imaging to explore the impact of L-dopa on gastric emptying [28]. The mechanisms postulated by which L-dopa delays gastric emptying were stimulations of dopamine and osmoreceptors [27]. It is also envisaged that it could also be a metabolite that may be responsible for delayed gastric emptying [27]; however, whichever it is, it affects both the absorption of L-dopa and its metabolite as this may also explicate the multiple peaks of methyldopa as well. Although the mean pharmacokinetic curve of IPB matrices has a single peak, some of individual pigs had double peaks and this may also explain the mean *T*_max_ of IPB matrices being at the 4th hour. Furthermore, the variability of gastric emptying is high and, apart from the presence of L-dopa, is an outcome of a complex interaction between the structure and function of the stomach and its nutrient content, which affects gastric emptying by meal volume and nutrient density. Gastric emptying is also affected by the physical and chemical properties of the meal, body movement, and position during emptying [30].

PKSolver, an add-in program for Microsoft Excel with user-friendly interface, predefined menus, and forms for easy recall [31], was used for computation. It is a visual basic for application (VBA) program which can run a range of applications for PK/PD data analysis including noncompartmental and compartmental analyses and modelling of pharmacodynamic data; and also embedded are 20 frequently used pharmacokinetic functions that can be executed on an open spreadsheet. PKSolver was validated by comparing its results with those of WinNonlin (Pharsight, Mountain View, USA) and Scientist (Micromath, Saint Louis, USA) employing two sample data sets obtained from a published book [31]. The parameters generated with PKSolver were similar to those obtained from WinNonlin and Scientist [31]. In fact, the results were identical to Scientist in all parameters to two decimal points and to WinNonlin to one or two decimal points. Consequently, PKSolver is not only flexible and user-friendly but also robust and reliable.

A noncompartmental pharmacokinetic model was chosen to decode the parameters for L-dopa plasma concentration-time curve as it best describes the data obtained (Table 8). The IPB matrices are characterized by higher *C*_max_,  *T*_max_,  AUC_0–*t*_,  AUC_0–inf_ and less apparent volume of distribution and clearance in comparison to Madopar HBS capsules and* PXLNET, *indicating a potential enhancement in the systemic bioavailability of L-dopa. The mean *T*_max_ of 4 hours for IPB matrices is attributed to the variations in individual pigs attaining peak plasma concentrations at different times. However, its mean residence time was decoded to be less than those of Madopar HBS capsules and* PXLNET. *

On application of ANOVA, the pharmacokinetic curves of the three formulations were found not to be statistically different (*p* = 0.49) at significance level of 0.05. *C*_max_ was also not statistically different (*p* = 0.44). Furthermore, when Madopar HBS capsules were compared with either IPB or PXLNET, there was no difference. However, statistical equivalence does not imply pharmaceutical equivalence or therapeutic equivalence. As modelled using the similarity factor, *f*_2_, the in vitro drug release profiles of IPB and* PXLNET *matrices were not bioequivalent to that of Madopar HBS capsules.

L-dopa is known to distribute widely into the body tissues while small amounts are found in the central nervous system. This is replicated and affirmed in this study by the large apparent volume of distribution for the three formulations and the small concentrations found in CSF. The volume of distribution when quantified per kilogram was 8.94, 15.09, and 11.20 L/kg for the IPB,* PXLNET*, and Madopar HBS, respectively. Furthermore, the large apparent volume of distribution may also clarify the low concentration of L-dopa in the plasma and appreciable concentration in urine when compared with plasma concentration. There was no urine data for* PXLNET *at the 4th hour as urine collection at that period proved abortive.

**Figure 9:**
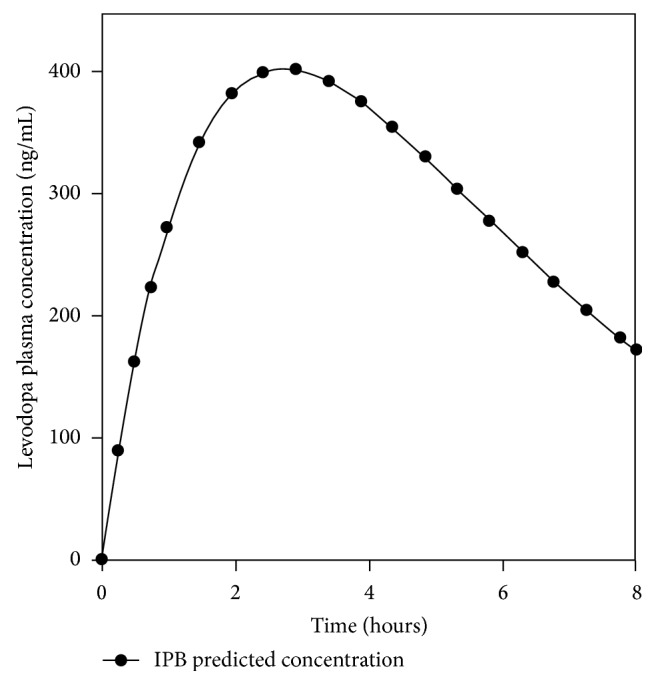
Predicted L-dopa plasma concentrations from 0.24 to 8 hours.

Based on the impracticality of continuous blood sampling throughout the day, from the observed values, the plasma concentrations for the time points samples that were not collected can be predicted.  Figure 9 is a predicted pharmacokinetic curve for IPB matrices for 8 hours showing the possible concentration of L-dopa for the times that samples were not collected. Obtaining predicted values is crucial in clinical situations as limited samples are collected after a dose to measure drug concentrations.

### 3.6. In Vitro-In Vivo Correlation of Dissolution and Pharmacokinetic Parameters

**Figure 10:**
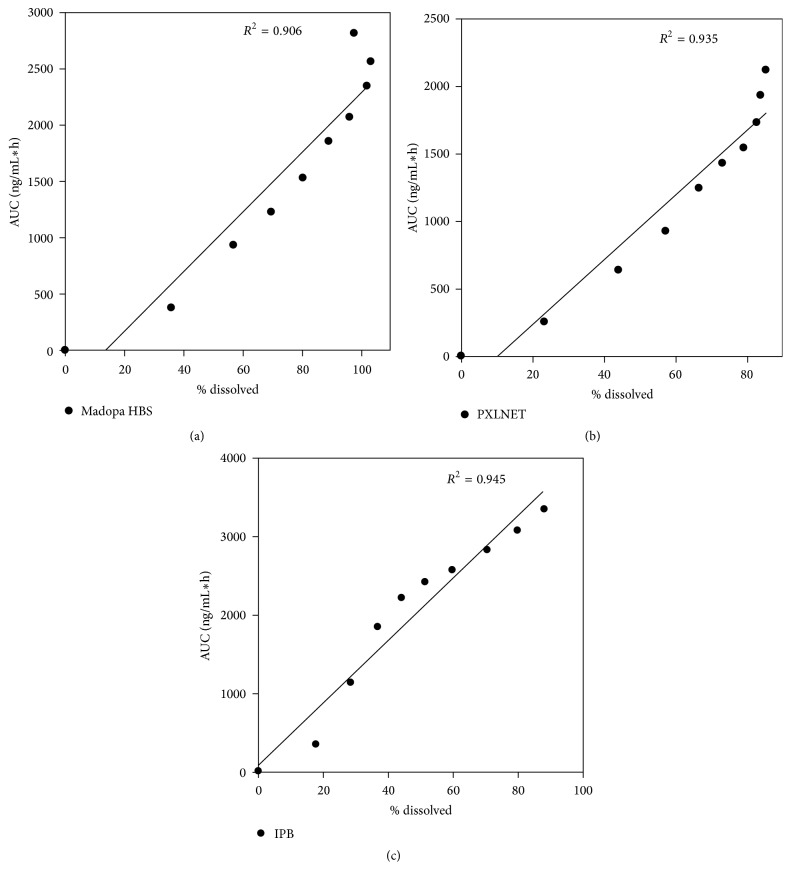
Linear regression multiple-level* C* IVIVC correlation models for (a) Madopar HBS capsules; (b)* PXLNET *matrices; and (c) IPB matrices.

In vitro-in vivo correlation describes the relationship between in vitro and in vivo outcomes. Various parameters can be used to assess correlations-dissolution time points such as* T*_50%_,* T*_90%_, MDT, and % dissolved for in vitro parameters and AUC, *C*_max_, and MRT for in vivo parameters. Of the five correlation levels, multiple-level *C* correlation was employed in this study. Multiple-level *C* correlation relates one or more pharmacokinetic parameters to the amount of drug dissolved (in vitro) at different time points of the dissolution profile [32]. In this study, a pharmacokinetic parameter, AUC was used to demonstrate a relationship with in vitro dissolution profile (% dissolved). A correlation is declared strong if it is greater than 0.8 and weak if it is less than 0.5. Linear regression multiple-level *C* IVIVC correlation models were constructed for Madopar HBS capsules,* PXLNET*, and IPB matrices and are shown in Figure 10. The correlation models had *r*^2^ values of 0.906, 0.935, and 0.945 for Madopar HBS capsules,* PXLNET*, and IPB matrices, respectively.

## 4. Conclusions

The IPB and PXLNET formulation has been proven in vivo to be gastroretentive and nontoxic to the tissues and cells. The pharmacokinetic parameters elucidated that L-dopa was liberated from the drug delivery systems, absorbed, widely distributed, metabolized and excreted as both unchanged and metabolites (such as methyldopa). The in vitro and in vivo data correlated strongly implying that quality gastroretentive drug delivery systems were developed which performs identically in vitro and in vivo. Therefore, IPB and* PXLNET *matrices designed and formulated show promise as gastroretentive drug delivery systems for delivery of L-dopa. Furthermore, the IPB GDDS potentially enhanced the systemic bioavailability of L-dopa compared to the market comparator, whereas the PXLNET achieved comparatively more notable CSF dopamine levels.

## Figures and Tables

**Table 1:** Multiple reaction monitoring parameters.

	Parent (*m/z*)	Daughter (*m/z*)
Dopamine	154.30	137.40
Levodopa	198.50	152.10
Methyldopa	212.90	165.90
Methyldopa	212.90	194.60
Carbidopa	226.40	181.1
Benserazide	258.70	139.10
Benserazide	294.70	258.70

**Table 2:** Measurement of dead cells (step 1).

Samples	Well 1	Well 2	Well 3	Well 4	Well 5	Well 6
10 mg nanoparticles	684	772	716	732	656	576
10 mg IPB	1474	1558	1428	1484	1466	1590
Cells only	970	1066	1014	1076	1228	960
Fresh medium	142	66	80	80	64	142
Empty wells	64	58	64	58	68	54

**Table 3:** Measurement of total cytotoxicity (step 2).

Samples	Well 1	Well 2	Well 3	Well 4	Well 5	Well 6
10 mg nanoparticles	778	744	834	924	832	868
10 mg IPB	1676	1662	1576	1414	1628	1836
Cells only	2784	2344	2810	2404	2576	2810
Fresh medium	118	108	102	92	80	118
Empty wells	150	164	148	172	144	116

**Table 4:** Signal from viable cells (step 2 − step 1).

Samples	Well 1	Well 2	Well 3	Well 4	Well 5	Well 6
10 mg nanoparticles	94	−28	118	192	176	292
10 mg IPB	202	104	148	−70	162	246
Cells only	1814	1278	1796	1328	1348	1850
Fresh medium	−24	42	22	12	16	−24
Empty wells	86	106	84	114	76	62

**Table 5:** Percentage cytotoxicity.

Samples	Well 1	Well 2	Well 3	Well 4	Well 5	Well 6
10 mg nanoparticles	5.18	−2.19	6.57	14.46	13.06	15.78
10 mg IPB	11.14	8.14	8.24	−5.27	12.02	13.30
Cells only	—	—	—	—	—	—
Fresh medium	—	—	—	—	—	—
Empty wells	—	—	—	—	—	—

**Table 6:** Mean cerebrospinal fluid concentration after oral administration of Madopar HBS capsules, *PXLNET*,and IPB matrices.

Time (h)	Mean CSF concentration (ng/mL)								
	Madopar HBS			*PXLNET*			IPB		
	M-D	D-M	L-D	M-D	D-M	L-D	M-D	D-M	L-D
2.00	36.47	—	88.69	35.63	83.01	70.48	3.27	—	71.96
4.00	28.31	—	224.12	27.86	82.94	87.79	30.44	—	97.15

M-D, methyldopa; D-M, dopamine; and L-D, levodopa.

**Table 7:** Mean urine concentration after oral administration of Madopar HBS capsules, *PXLNET*, and IPB matrices.

Time (h)	Mean urine concentration (ng/mL)								
	Madopar HBS			*PXLNET*			IPB		
	M-D	D-M	L-D	M-D	D-M	L-D	M-D	D-M	L-D
2.00	604.6	5888	196.8	1532.7	4449	784.8	459.4	111.2	425.5
4.00	853.3	21938	755.1	—	—	—	1221.8	4571.1	1328

M-D, methyldopa; D-M, dopamine; and L-D, levodopa.

**Table 8:** Levodopa noncompartmental pharmacokinetic parameters following oral administration of Madopar HBS capsules, *PXLNET*, and IPB matrices.

Pharmacokinetic parameter	Madopar HBS	*PXLNET*	IPB
*T*_max_ (h)	2	2	4
*C*_max_ (ng/mL)	372.37	257.02	461.28
AUC_0–*t*_ (ng/mL*∗*h)	2816.47	2121.43	3347.45
AUC_0–inf_ (ng/mL*∗*h)	3685.03	2722.42	4147.16
AUMC_0–inf_ (ng/mL*∗*(h)^2^)	56590.17	40775.35	55147.93
MRT (h)	15.36	14.98	13.30
Vz/F [mg/(ng/mL)]	0.3598	0.4847	0.2874
Cl/F [mg/(ng/mL)/h]	0.0271	0.0367	0.0241

*T*_max_, time for maximum concentration of drug; *C*_max_, maximum drug concentration; AUC_0–*t*_, area under the concentration-time curve; AUC_0–inf_, area under the concentration-time curve from time 0 to infinity; AUMC_0–inf_, area under the first moment of concentration-time curve from time 0 to infinity; MRT, mean residence time. Cl/F is apparent clearance and Vz/F is apparent volume of distribution.
